# Safety and efficacy of aortic coarctation stenting in children and adolescents

**DOI:** 10.1016/j.ijcchd.2022.100389

**Published:** 2022-05-11

**Authors:** Biagio Castaldi, Elena Ciarmoli, Angela Di Candia, Domenico Sirico, Giuseppe Tarantini, Fabio Scattolin, Massimo Padalino, Vladimiro Vida, Giovanni Di Salvo

**Affiliations:** a Biagio Castaldi Pediatric Cardiology Unit, Department of Women's and Children's Health, University of Padua, Via Giustiniani 3, 35129, Padova, Italy; b Department of Pediatrics, ASST Brianza, Vimercate Hospital, Italy; c Department of Cardiac Thoracic and Vascular Sciences and Public Health, University of Padua, Italy

**Keywords:** Aortic coartctation, Stent, Congenital heart disease, Interventional cardiology, Catheterization

## Abstract

Percutaneous aortic coarctation treatment with primary stent implantation is the first choice in adult patients. However, current guidelines do not suggest a lower weight limit to perform this procedure safely.

The aim of this study was to retrospectively analyze the safety and the mid-term outcome of aortic coarctation stenting in pediatric age at different patients’ weights.

We enrolled 47 patients, 8 of them weighting lower than 25 kg, 10 with a weight between 25 and 30 kg and 29 patients with a weight >30 kg.

Covered CP stent was used in 32 patients (68.1%), bare CP stent in 6 (12.8%), Andrastent XL in 8 (17.0%) and Palmaz Genesis in one case (2.1%). The balloon mean diameter was 13.8 ± 2.4 mm, range 10–18 mm. The procedure was effective in all patients. The aortic gradient dropped from 28.0 ± 7.8 mmHg to 2.2 ± 2.0 mmHg (p < 0.0001). Hemostasis was achieved by a surgical cut-down in 20 (42.5%) patients, manual compression in 2 (4.3%) or by vascular closure devices (VCDs) in 25 (53.2%) patients. There was no difference in terms of efficacy, safety and complication rate among the three weight-based groups. We found a trend toward higher incidence of vascular complications following hemostasis with VCDs (4/24, 16.7%) vs surgical cut-down (1/21; 4.8%)

In conclusion, aortic coarctation stenting is a safe procedure in patients weighing less than 30 kg. Surgical arterial cut-down can minimize the risk of vascular injury by reducing the stress on the arterial wall in smaller patients, or in case, a large sheath is needed.

## Highlights

• There are few evidences regarding the lower weight limit suitable for a safe stent implantation in aortic coarctation.
• Surgical cut-down may reduce vascular complications in smaller patients.
• An elective surgical vascular cut-down allows a safe *trans*-catheter treatment below the 20 kg.

## 1 Introduction

Aortic coarctation accounts for about 7% of known congenital heart defects with a frequency of about 0.04% of live births [1]. The prevalence of coarctation is increased in some genetic disorders, such as Turner syndrome [2]. In addition, it is commonly associated with cardiac bicuspid aortic valve (up to 85%) [3].

The use of bare metal stent for treatment of aortic coarctation was described for the first time in 1991 [4]. This procedure has become progressively a widely performed intervention, proving safe and effective treatment for either native lesion or recurrence after surgical repair [[5], [6], [7], [8], [9]]. Covered stents were firstly described to treat vessel complications [[10], [11], [12]], and progressively become the first-choice treatment in patients at higher risk of aortic wall complications [1,13,14].

Current ESC and AHA guidelines indicate stent implantation as first-choice treatment for native or recurrent aortic coarctation in adults [3,15]. Conversely, specific guidelines for pediatric coarctation treatment are lacking. AHA recommendations for cardiac catheterization and intervention in pediatric cardiac disease only suggest the percutaneous approach in suitable anatomies and when the implantable stent can be expanded to an adult size [16]. Finally, there is scarce evidence and no guidelines regarding the lower weight and/or age limit suitable for a safe percutaneous stent implantation in aortic coarctation.

The aim of this study was to retrospectively analyze the safety and the mid-term outcome of aortic coarctation stenting in pediatric age at different patients’ weight.

## 2 Methods

From 2012 to 2019, 47 patients with isolated aortic coarctation underwent percutaneous stent implantation for native or recurrent aortic coarctation at our Institution. The study was performed in agreement with the advices of the Helsinki Declaration. Patients with genetic syndromes and with complex congenital heart diseases (e.g. single ventricle physiology, Norwood procedure) were excluded from the study. The indications to aortic coarctation treatment were: blood pressure gradient between upper and lower limbs greater than 20 mmHg at rest, or less than 20 mmHg associated with systemic arterial hypertension at rest and left ventricular dysfunction or hypertrophy. Percutaneous stent implantation was considered if the weight was greater than 25 kg, or lower than 25 kg in case of favorable aortic anatomy on cardiac MRI assessment and in presence of common femoral artery diameter larger than 4 mm on ultrasound assessment. Patients were divided in three groups based on the weight at the time of the procedure: lower than 25 kg (Group A), between 25 and 30 kg (Group B) and greater than 30 kg (Group C).

The procedures were performed in general anesthesia. Informed consent was obtained from all patients or their parents prior to the procedure. Intravenous heparin (100 IU/kg, maximum of 5000 IU) and Aspirin (5 mg/kg, maximum 300 mg) were given immediately after femoral artery cannulation.

The femoral access was obtained with Seldinger technique and a 5 Fr sheath was used for diagnostic catheterization in order to confirm the diagnosis, define the anatomy and to choose the type and size of the stent. The stents used were bare or covered Cheatham-Platinum (CP) stents (NuMed, Hopkinton, NY), Andrastent (Andramed GmbH, Germany) and Palmaz Genesis stents (Cordis Corporation, Miami, FL). In particular, covered stents were used in tight lesions, in case of associated ascending aorta dilatation or when the angiography showed isthmic additional lesions like aneurisms or patent ductus arteriosus. The stents were manually crimped on the balloon catheter: Atlas, Atlas Gold (Bard, Tempe, AZ) or XXL (Boston Scientific, Marlborough, MA) balloon catheters. The diameter of the balloon was chosen based on the transverse arch diameter, while the balloon length was selected as the first size longer than the stent length. Once the stent was crimped, we chose the smallest Mullins long sheath able to contain the crimped stent. The procedure was completed through the same vascular access or through the counter-lateral femoral artery after surgical arterial cut-down. The choice between percutaneous versus surgical approach was based on the sheath size, the weight of the patient and the angiographic features of the iliac and femoral arteries. At the end of the procedure, the hemostasis was achieved by manual compression or by vascular closure devices (VCDs) as Prostar XL (Abbott, Chicago, IL), Perclose ProGlide® (Abbott, Chicago, IL) or Angioseal® (St Jude Medical, St. Paul, MN) in case of percutaneous access and by surgical repair in case of surgical cut-down.

All patients were monitored for vascular access related complications with groin auscultation and assessment of peripheral pulses and ultrasound evaluation 24 h after the procedure. Antibiotic prophylaxis for 24 h and aspirin 100 mg OD for 1 month after the implantation were administered to all patients.

Complications were classified as major or minor as follows. Events requiring surgical treatment or determining chronic sequelae were identified as major events (e.g. femoral artery thrombosis or dissection requiring surgical treatment, detection of femoral artery thrombosis at the follow up). Transitory events requiring no treatment or temporary drug infusion (i.e. intravascular nitrate, or an additional dose of heparin) were considered minor events (e.g. femoral artery spasm, arrhythmias).

Outpatient follow-up consisted of clinical assessment including four-limb blood pressure and need for antihypertensive medications, ECG and transthoracic echocardiogram at 6, 12 months and then yearly.

### 2.1 Statistical analysis

The normal distribution of the data was assessed by Kolmogorov–Smirnov's test. Quantitative values are reported as mean + 1 SD. Univariate analysis was performed using the χ2 test, Fisher's exact test, unpaired Student's t-test, Wilcoxon rank sum test, and paired *t*-test as appropriate. The comparison between the three group was performed with ANOVA and Bonferroni Post-hoc analysis. Multi-variable analysis was performed using multiple logistic regression analysis to study risk factors associated with major complications. Independent variables with a P-value <0.05 in the univariate analysis were included in the multivariable model. Odds ratios and their 95% confidence intervals were calculated for independent variables included in the multivariable model. The null hypothesis was rejected for a P-value <0.05. All analyses were performed by using a commercially available package (SPSS Inc., Chicago, IL, v. 26.0).

## 3 Results

Forty-seven patients were enrolled in this study (Table 1). The mean age was 12.2 ± 4.6 years (range 4–20), the mean weight 42.3 ± 16.8 kg. Ten patients (21%) were on anti-hypertensive therapy: beta-blockers in 4, ACE-inhibitors in 3, diuretics in 2 and Calcium channel blockers in 1. The association with a bicuspid aortic valve was present in 34 (73%) patients. The coarctation was native in 39 patients while was a post-surgical recurrence in eight. Associated lesions were: ventricular septal defect in 7 patients; mitral valve stenosis in one patient, mitral valve regurgitation in one, atrial septal defect in one, and atrioventricular septal defect in one patient.

**Table 1:** General characteristics of the population study and subgroup analysis. Data are expressed as mean value ± standard deviation (range) or number (%).

	Total	Patient groups based on body weight		
		Group A (<25 kg)	Group B (25–30 kg)	Group C (>30 kg)
N° of patients	47	8 (17%)	10 (21.3%)	29 (61.7%)
Age (years)	12.0 ± 4.8 (4–20)	6 ± 2 (3–8) °^	9 ± 1 (7–11)*	15.6 ± 3.0 (9–20)
Weight (kg)	42.3 ± 16.8 (18–76)	21 ± 3 (18–25) °^	29 ± 1 (30–27)*	52 ± 12 (32–76)
Height (cm)	147 ± 20 (106–176)	116 ± 8°^ (106–130)	135 ± 6* (127–143)	160 ± 12 (128–176)
SBP (mmHg)	131 ± 15 (101–162)	115 ± 6°^ (107–125)	125 ± 16* (101–152)	137 ± 12 (120–162)
Patients on anti-hypertensive therapy	10 (21%)	1 (12,5%)	0	9 (31%)
Distal transverse aortic arch diameter (mm)	15 ± 5 (9–31)	11 ± 1 (10–12) °^	13 ± 3 (9–17)*	16 ± 5 (10–31)
Minimum diameter at isthmus (mm)	7 ± 3 (2–13)	4 ± 1 (3–5) °^	7 ± 2 (4–11)	7 ± 3 (2–13)
Aortic diameter at diaphragm (mm)	14 ± 4 (8–22)	10 ± 2 (8–14)	13 ± 4 (8–20)	16 ± 3 (10–22)
Sheath size (French)		°	*	
- 8	1 (2.1%)	0	1 (10%)	0
- 9	2 (4.2%)	2 (25%)	0	0
- 10	9 (19.1%)	3 (37,5%)	2 (20%)	4 (13.8%)
- 11	8 (17%)	2 (25%)	3 (30%)	3 (10.3)
- 12	21 (44.7%)	1 (12,5)	4 (40%)	16 (55.2%)
- 13	1 (2.1%)	0	0	1 (3.4%)
- 14	5 (10.6%)	0	0	5 (17.2%)
Surgical isolation	21 (44.6%)	5 (62.5%)	4 (40%)	12 (41.3%)
Hemostatic device	24 (51.0%)	2 (25%)	6 (60%)	16 (55.1%)
Manual compression	2 (4.3%)	1 (12,5%)	0 (0%)	1 (3.4%)
Major vascular complication	5 (10.6%)	2 (25%)	0	3 (10.3%)
Minor vascular complication	9 (19.1%)	5 (62.5%)°	2 (20%)	2 (6.9%)
Stent				
- covered CP stent	32 (68.1%)	5 (62.5%)	8 (80%)	19 (65.5%)
- bare CP stent	6 (12.7%)	1 (12.5%)	1 (10%)	4 (13.8)
- Andra XL	8 (17.0%)	2 (25%)	0	6 (20.7%)
- Palmatz Genesis	1 (2.1%)	0	1 (10%)	0
Balloon angioplasty diameter (mm)	13.8 ± 2.4 (10–18)	11 ± 1 (10–12) °^	12 ± 1 (10–14)*	15 ± 2 (12–18)
Pre-procedure aortic gradient (mmHg)	28.0 ± 7.8 (11–40)	29 ± 7 (21–35)	30 ± 6 (20–40)	27 ± 8 (11–40)
Post-procedure aortic gradient (mmHg)	2.2 ± 2.0 (0–16)	3.6 ± 5.6 (0–16)	1.8 ± 3.4 (0–10)	1.7 ± 2.8 (0–10)
Procedural time (min)	92.30 ± 26.62 (60–150)	92.50 ± 28.57 (60–150)	82.50 ± 28.72 (60–120)	95.42 ± 26.30 (60–150)
Fluoroscopy time (min)	13.53 ± 4.89 (6.0–23.0)	11.88 ± 4.08 (7.0–17.0)	12.66 ± 4.78 (8.1–17.0)	14.3 ± 5.29 (6.0–27.0)
DAP (Gy*cm^2^)	25.90 ± 22.02 (3.90–90.00)	9.56 ± 5.56° (3.9–16.6)	9.03 ± 6.92 * (2.08–20.0)	37.76 ± 21.68 (4.40–90.00)
Medium of Contrast (cL)	148.4 ± 51.7 (80–200)	111.0 ± 43.1° (80–150)	133.7 ± 45.3 (100–180)	179.4 ± 43.3 (110–260)

SBP: systemic blood pressure.

° p < 0.05 group A vs Group C.

*p < 0.05 group B vs Group C.

^p < 0.05 group A vs Group B.

**Fig. 1:**
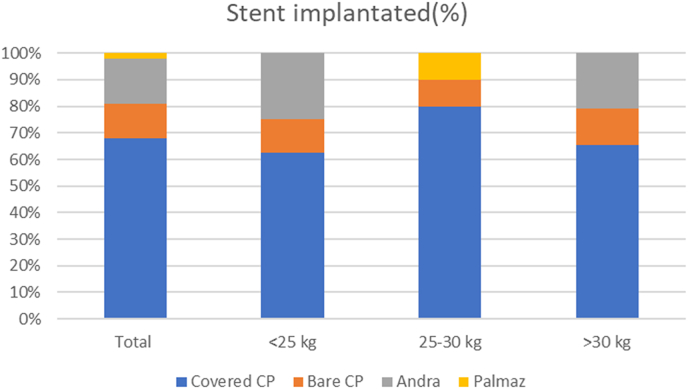
Percent of stent implanted in the three groups.

Covered CP stent was used in 32 patients (68.1%), bare CP stent in 6 (12.8%), Andrastent XL in 8 (17.0%) and Palmaz Genesis in one case (2.1%) (Fig. 1). The balloon diameter used was 13.8 ± 2.4 mm, range 10–18 mm. The procedure was effective in all the patients. The invasive aortic gradient dropped from 28.0 ± 7.8 mmHg to 2.2 ± 2.0 mmHg (p < 0.0001).

**Fig. 2:**
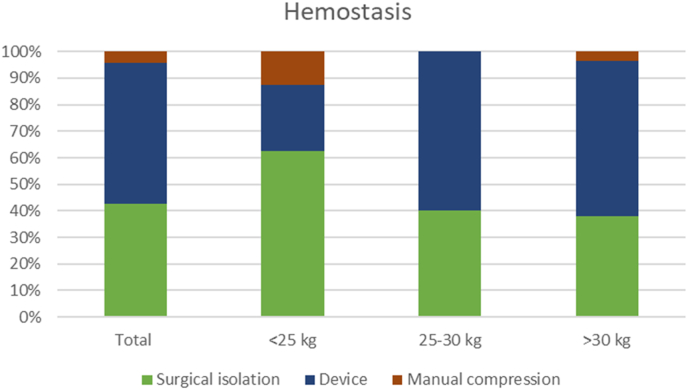
Techniques used for hemostasis in the three groups.

A surgical cut-down and closure of the vessel was performed in 21 (44.6%) patients, manual compression in 2 (4.3%), while VCDs were used in 24 (51.0%) patients, (Prostar XL in 18 cases, ProGlide in 4 patients, Angioseal in 3) (Fig. 2).

No acute complication occurred on the coarctation site. On the other hand, five major vascular complications occurred on the vascular access: 4 femoral artery and/or external iliac artery dissection and one femoral artery thrombosis, four of them requiring surgical repair and/or thrombectomy. In addition, transient spasm of the ileo-femoral arterial axis was evident in 9 (18.7%) patients.

### 3.1 Subgroup analysis

There were eight patients in group A, 10 patients in group B, and 29 patients in group C. There was no difference in terms of efficacy, safety and complication rate among the three groups. The three groups differed regarding anthropometric data, aortic diameters, balloons and sheath sizes, according with the weight at the time of the procedure (Table 1). No difference was found in terms of procedural and fluoroscopic times. On the other hand, DAP and medium contrast volumes were higher in group C.

### 3.2 Vascular complication analysis

There was trend towards higher vascular complications among group A. Specifically, complications occurred in two patients out of eight (25%): an 18-kg patient in whom the 10 Fr vascular access was closed with an Angioseal device (complication: late diagnosis of femoral artery thrombosis with effective collateralization), and an 18.4-kg patient with surgical cut-down with positioning of a 12 Fr Mullins Sheath (complication: external iliac artery rupture, requiring surgical bypass). No complications occurred in group B, while 3 (10.3%, n = 29) major complications occurred in group C. (arterial thrombosis in a 38-kg patient, 12 Fr Sheath, hemostasis with Prostar; femoral artery dissection requiring surgical treatment in two cases: 10 Fr sheath in a 61-kg patient, hemostasis with Proglide; 14 Fr sheath in a 68-kg patient, hemostasis with Proglide).

Furthermore, vascular complications following hemostasis with a device (4/24, 16.7%) were slightly higher, when compared with surgical cut-down (1/21; 4.8%) and manual compression (0/2), although this finding did not reach statistical significance (p = 0.36) (Table 2).

**Table 2:** Comparison between hemostatic Device and surgical isolation of the vascular access.

	Hemostatic device	Surgical isolation
N° of patients	25	20
Age (years)	12.5 ± 4.8	12.9 ± 7.3
Weight (kg)	44.4 ± 15.8	40.1 ± 17.3
Istmus diameter (mm)	6.6 ± 2.8	6.7 ± 2.7
Sheath size (French)	11.7 ± 1.2	10.9 ± 1.6
Balloon diameter (mm)	14.3 ± 2.6	13.4 ± 2.2
Major Complications	4	1
Minor Complications	3	4

Univariate analysis did not find any correlation between age, weight, Mullins sheath size and closure system and major vascular complications. Minor vascular complications were influenced by age at intervention (R = −0.295; p = 0.017); weight (R = −0.297; p = 0.014) and Mullins sheath size (R = 0.384; p = 0.004).

Multivariate analysis showed that minor vascular complications were associated to the patient's weight (F = 5.52; p = 0.023) and to the Mullins sheath size (F = 11.76; p = 0.001). Finally, when analyzing any vascular complication (minor and major), the only variable that reached a statistical significance was the Mullins sheath size (F = 6.54, p = 0.003).

### 3.3 Follow-up

The mean follow-up was 4.2 ± 3.1 years (range 1–10 year, median 4.1 years). The mean isthmic gradient by echo was 13.1 ± 6.8 mmHg, it was higher than 20 mmHg in 4 patients (two in group A and two in the group C). Percutaneous balloon stent re-dilation was performed in 2 (25%) patients of the group A, 3 years and 4 years after the first procedure; in one patient (10%) in group B 6 years after the first procedure.

In two cases a stent fracture was detected (1 Palmaz Genesis, 1 Andrastent XL) and the patient treated with re-stenting with a covered CP stent. In one patient with a basal complex lesion including aortic wall aneurism, the follow-up CT scan showed a persisting aortic aneurism, treated successfully with a second covered stent placement. One year after the procedure, 6 patients (12.8%) were still on hypertensive mono-therapy (3 on beta-blockers, 3 on ACE-Inhibitors). The remaining 41 patient showed normal systolic pressure by office evaluation and 24 h monitoring. The systolic pressure was 122.6 ± 11.3 mmHg (mean age at evaluation 19.2 ± 5.7 years), without any difference between the three groups (Group A: 120.3 ± 13.0 mmHg, age 13.2 ± 1.6 years; Group B: 118.5 ± 9.2 mmHg, 15.3 ± 2.4 years; Group C: 126.3 ± 11.4 mmHg, 22.2 ± 5.1 years at the last follow-up).

## 4 Discussion

Stent implantation in aortic coarctation is the treatment of choice in adolescent and adult patients [3,15,16]. This procedure helps to stabilize the vessel's diameter after balloon dilation and maintains the effectiveness regardless of intimal injury [7,17] reducing the risk of restenosis. However, guidelines do not indicate a lower weight or age cut-off to proceed with stent implantation.

Although aortic balloon angioplasty is an option in infants with recurrent aortic coarctation after surgical repair, aortic stenting is the currently recommended first choice treatment in either native or recurrent coarctation in pediatric and adult age. Guidelines recommend implantation of stents dilatable to adult size diameters [16], as a consequence the most employed options are CP stent, AndraStent and Palmaz Genesis stent. The latter requires at least an 8 Fr long sheath, AndraStent requires at least a 9 Fr sheath, bare CP stent requires at least a 10 Fr sheath while for a covered CP stent at least an 11 Fr sheath (pre-mounted 12 Fr) is needed. Therefore, the main issue in this procedure is the adequacy of the vascular access.

In our retrospective study, surgical cut-down and percutaneous puncture followed by VCDs implantation were commonly used techniques. From 2012 to 2015, VCDs represented the most frequent choice for hemostasis (24/27 patients), however 3 out of 4 major complications occurred following hemostasis with these devices. From 2016 to 2019, surgical cut-down and subsequent repair constituted the technique of choice (17/20 patients), and the only major vascular complication occurred in a patient treated with a VCD. Probably the borderline diameter of femoral artery and the high incidence of arterial spasm during this type of procedure might explain the relatively high incidence of vascular lesions in this cohort of patients [18]. Conversely, surgical cut-down of the vessel permits a direct viewing of the vascular access, so that the operator can appreciate the degree of tension applied to the femoral artery and modulate the traction force needed for the sheath removal. In addition, this technique may be able to eliminate the friction given by the other anatomical layers (muscles, sub-cutis and skin) on the sheath during the maneuver. Finally, the arterial angiography of the ileo-femoral axis from the counter-lateral arterial access allows early identification and correction of residual defects on the arterial wall after Mullins sheath removal. Hence, in pediatric age, we currently consider surgical cut-down the first-choice vascular access for aortic coarctation stenting when a large sheath is needed.

There are few and small studies aiming the effectiveness and the safety of aortic coarctation stenting in small patients [[19], [20], [21], [22], [23]]. In these papers, low profile stents were often preferred to CP stent in order to minimize the sheath size. However, these stents (i.e. Palmaz, Formula, Valeo and Mega LD) are peripheral vascular stents (off label for this procedure), and in some cases no more available on the market. In addition, the risk of stent fracture is not negligible and has been reported for these stents, in particular Palmaz Genesis [[24], [25], [26], [27]]. On the other hand, these stents are safer on vascular injuries, can be re-dilated to adult sizes or even intentionally fractured to re-stent with a larger device [22]. In our study, a peripheral vascular stent was used in one case, followed by stent fracture, re-coarctation and necessity of re-stenting. On the other hand, CP and Covered CP stents were effectively used from 18 kg (5/8 covered CP stent in group A, 8/10 covered CP stents in group B). In these cases, the sheath was flushed and poured with nitroglycerine to minimize the risk of arterial spasm or dissection. At the end of the procedure, a slow and gentle retrieval of the Mullins sheath was facilitated with purges of saline and nitroglycerine from the sheath or at the common iliac artery origin by using a 4 Fr Rim catheter from the counter lateral femoral artery under invasive arterial pressure monitoring.

In our population, the number of covered stents was higher compared to similar series. The choice of bare versus covered stent should be carefully evaluated with a non-invasive pre-operative comprehensive assessment in order to plan the procedure and to assess the risk of vascular injuries, especially in small patients. In patients <30 kg with borderline anatomies, we preferred a primary covered stent implantation because a rescue procedure, including a Mullins sheath replacement from the same femoral artery, can be extremely difficult in case of arterial spasm. On the other hand, when the anatomy is favorable for a bare metal stent approach, an elective surgical vascular cut-down allows a safe *trans*-catheter treatment below the 20 kg.

## 5 Limitations

The retrospective nature of the present study may represent a major limitation; in fact, the type and size of stents implanted were selected by the cath lab team and did not follow a designed protocol. In addition, we could not compare the results with a control group (e.g. alternative techniques, surgically treated cohort).

The follow-up of these patients was routinely performed at our outpatient clinic department, with clinical and echocardiographic assessment. Chest X-ray, angioCT and/or angioMR were performed in selected cases when stent dysfunction or size mismatch with a transverse arch diameter were suspected.

A longer follow-up may demonstrate a higher rate of re-intervention for stent re-dilation, especially in the smallest group.

## 6 Conclusion

Aortic coarctation stenting is a safe procedure in pediatric patients weighing less than 30 kg. Surgical arterial cut-down might minimize the risk of vascular injury in smaller patients or in case a large sheath is needed. In presence of favorable anatomy for a bare metal stent implantation, an elective percutaneous approach may be considered below the 20 kg.

## Disclosure

Authors have no source of funding to declare.

## Declaration of competing interest

No conflict of interest.
